# Factors affecting test anxiety: a qualitative analysis of medical students’ views

**DOI:** 10.1186/s40359-021-00715-2

**Published:** 2022-01-06

**Authors:** Majed Wadi, Muhamad Saiful Bahri Yusoff, Ahmad Fuad Abdul Rahim, Nik Ahmad Zuky Nik Lah

**Affiliations:** 1 Medical Education Department, College of Medicine, Qassim University, Buraidah, Saudi Arabia; 2 Medical Education Department, School of Medical Sciences, Universiti Sains Malaysia, Kota Bharu, Kelantan Malaysia; 3 Obstetrics and Gynecology Department, School of Medical Sciences, Universiti Sains Malaysia, Kota Bharu, Kelantan Malaysia

## Abstract

**Background:**

Medical students are vulnerable to test anxiety (TA), which impacts their professional lives and jeopardizes the optimal health care of their patients. The qualitative exploration of TA among medical students is crucial to understanding the problem. Hence, this study examined medical students’ insights into TA and their suggestions on how to reduce it.

**Methods:**

We conducted a phenomenological study on medical students at a public university. We utilized focus group discussions (FGDs) to investigate their experiences of TA. The FGDs were transcribed verbatim, and these transcripts were analyzed using Atlas.ti software. The thematic analysis followed the recommended guidelines.

**Results:**

Seven FGD sessions were conducted with 45 students. Three major themes emerged: the students, their academic resources, and the examiner. Each theme comprised mutually exclusive subthemes. The “students” theme was divided into negative vs. positive thoughts and self-negligence vs. self-care, “academic recources” into heavy curriculum vs. facilitative curricular aids, and “examiner” into criticism vs. feedback and strict vs. kind approaches.

**Conclusion:**

This study provides a solid foundation for policymakers and decision makers in medical education to improve current assessment practices and student well-being. Medical students will be able to significantly alter and reduce TA if they are provided with additional psychological support and their examiners are trained on how to deal with examinees.

## Introduction

Test anxiety (TA) is integral to assessments. It ranges from simple worry to debilitating anxiety, which interferes with the cognitive process. Although a low level of TA can motivate students to study and prepare for assessment, an extreme level of TA alters their physiological functions, psychological status, or both. These changes impair concentration, interrupt working memory, and hinder academic achievement. Crucially, TA may result in chronic stress, which is associated with many adverse effects on wellbeing. These include burnout, depression, poor academic performance, poor clinical performance, impaired decision making, poor peer interaction, interpersonal conflict, academic dishonesty, and sleeping problems [1, 2]. Furthermore, chronic stress is linked to substance abuse, alcohol consumption, and suicide [3–6].

TA has a significant effect on health professions’ students. In a meta-analysis, Quek, Tam [7] reported that 33.8% of medical students experience anxiety. Likewise, Macauley, Plummer [8] determined that 51% of female and 37.5% of male health care students have moderate to high TA. Several quantitative studies have explored the causative factors of TA among medical students. These factors included expansive curricular content [9–12], inappropriate study skills [9–12], difficult test formats (objective structured clinical examination [OSCE] in particular), negative thoughts [10], and female gender [9, 10, 12]. However, the few qualitative studies that have examined TA among medical students are limited in scope. For instance, Encandela et al. [13] assessed TA following the implementation of a United States Medical Licensing Examination (USMLE) preparation course, whereas Shen et al. [14] explored whether the introduction of expressive writing can reduce TA.

Qualitative research yields detailed insights, sheds light on the dynamics of various relationships, and generates themes and thus theoretical foundations for future research [15]. Therefore, the qualitative investigation of medical students’ TA is essential to understanding its implications. Such studies can clarify how to remodel TA and create a platform for early intervention into and improvement of medical students’ well-being. Hence, this qualitative study explored medical students’ thoughts on TA and what they believed should be done to reduce it.

## Methods

### Approach

This study employed a phenomenological approach to analyze the factors affecting TA from the perspectives of medical students. Data were collected using seven focus group discussions (FGDs) with a total of 45 medical students from the School of Medical Sciences, Universiti Sains Malaysia (USM). The study was approved by the Institutional Human Research Ethics Committee at USM, and all students were asked to sign a consent form upon their agreement to participate in this study. The researchers emphasized confidentiality, anonymity, and the right to withdraw at the start of each session. To maintain the participants’ confidentiality, we assigned a pseudonym to each one.

### Participants, sampling, and recruitment

We selected groups of five to seven medical students from different academic years because we expected their experiences of stress and anxiety to differ accordingly [16]. We applied purposive sampling with consideration for student variety to obtain a wide range of experiences. Moreover, we intended to represent factors such as gender and race (e.g., Malay, Chinese, Indian, or other). We gave invitation letters to the group leaders of each academic year. We also used WhatsApp to disseminate information about the study and its consent forms. In addition, we assigned a token to each participant after their session.

### Data collection

We piloted the FGD protocol with a group of students, and they reported that it was open-ended and stimulated discussion. Next, the FGD sessions were conducted in a quiet and comfortable room. We began each session by welcoming the group members and briefing them on the study’s purpose. Based on the predetermined probe questions, we initiated the audiotaped discussion with an open-ended cue: “Test anxiety to me is…”. We made notes to reflect non-verbal cues. Each FGD ended after 60 to 90 min. Data collection continued until theoretical saturation was reached, which occurred when no new information appeared [17]. We conducted all the FGD sessions in March 2019.

### Data analysis

We started data analysis concurrently with data collection. This interim analysis helped us adjust and check the emerging themes alongside the consequent data.

We followed Braun and Clark’s six-phase thematic analysis [18]. In the first phase (familiarization with the data), the researcher (MW) transcribed the audio recording verbatim (including verbal and non-verbal cues), assigned pseudonyms to all the identifiable individuals, and cross-checked the transcript against the audio recordings. All the authors (MW, MSBY, AFAR, and NAZNL) then read the transcription several times to familiarize themselves with the data set and to immerse themselves in its meaning. Then, MW imported all the transcription files into Atlas.Ti (version 7.9) to initiate the second phase (generating initial coding), during which MW and MSBY independently identified open codes throughout the data set; these were either the participants’ own words (in vivo) or a descriptive word for their experience. We conducted frequent comparisons of the generated themes to resolve disagreements and reach consensus on the initial themes. In phase three (searching for themes), MW and MSBY conducted a high-level analysis by combining several related codes to create overarching themes. MW, MSBY, AFAR, and NAZNL held joint meetings to discuss potential themes. In the fourth phase (reviewing the themes), MW examined the quotations associated with each theme and determined their coherence (internal homogeneity). If a quotation did not fit, MW either redirected it to a more closely related theme or revised the theme. MW then reviewed all the themes to determine their relevance and to ascertain whether each theme was significantly different from the others (external heterogeneity). To ensure that potential themes reflected the entire data set, MSBY and AFAR compared them to the codes and to the entire data set. MW, MSBY, AFAR, and NAZNL discussed inconsistencies and refined potential themes. In the fifth phase (defining and naming themes), we gave each a name, a definition, and an explanation narrative. Additionally, MW, MSBY, AFAR, and NAZNL ascertained whether any complex theme required subthemes to be structured more effectively. Finally, in the sixth phase (writing the report), MW and AFAR assembled the selected quotes to illustrate key points. MSBY and NAZNL revised the report. We repeated this procedure until we reached unanimous agreement.

Table 1 highlights how we addressed Guba’s four criteria for detecting the trustworthiness of qualitative studies [19].

**Table 1:** Provisions made to address the findings’ trustworthiness [19]

Aspect of trustworthiness	Implementation process
Credibility (internal validity)	**Trained moderator** All researchers received training in qualitative research; additionally, the principal researcher (MW), who serves as the moderator of the FGD in this study, conducted a pilot FGD prior to conducting the actual data collection **Prolonged engagement** During the FGD, MW ensured that participants had ample opportunity to share their experience **Data source triangulation** We employed methodological triangulation in our study, in which we compared the qualitative findings to the quantitative findings **Member checking** We used two strategies for member checking: (1) iterative questioning by extracting related data via rephrased questions “on the spot,” and (2) summarizing the key points of discussion and asking participants to verify the summary while also allowing for additional remarks
Transferability (external validity)	**Thick descriptions** The method section includes a detailed description of the data setting, informants, and phenomena, which enables future researchers to transfer the findings
Dependability (reliability)	**Code-recode procedure** The researchers (MW and MSBY) used a code-recode technique in which they returned to previously coded raw data to recode it and compare it to the previous coding. They used this method two weeks after the initial coding to ensure that the results were consistent
Confirmability (objectivity)	**Audit trail** MW kept a detailed account of the FGD files and analysis process for the audit

## Results

Table 2 displays the characteristics of the participants. Gender distribution was nearly equal among participants, and most were fourth-year students.

**Table 2:** Characteristics of the participants

Variable		
Gender	Male	23 (51%)
	Female	22 (49%)
Year of study	2nd year	8 (18%)
	3rd year	16 (36%)
	4th year	21 (47%)
Race	Malay	22 (49%)
	Chines	11 (24%)
	Indian	9 (20%)
	Others	3 (7%)

**Fig. 1:**
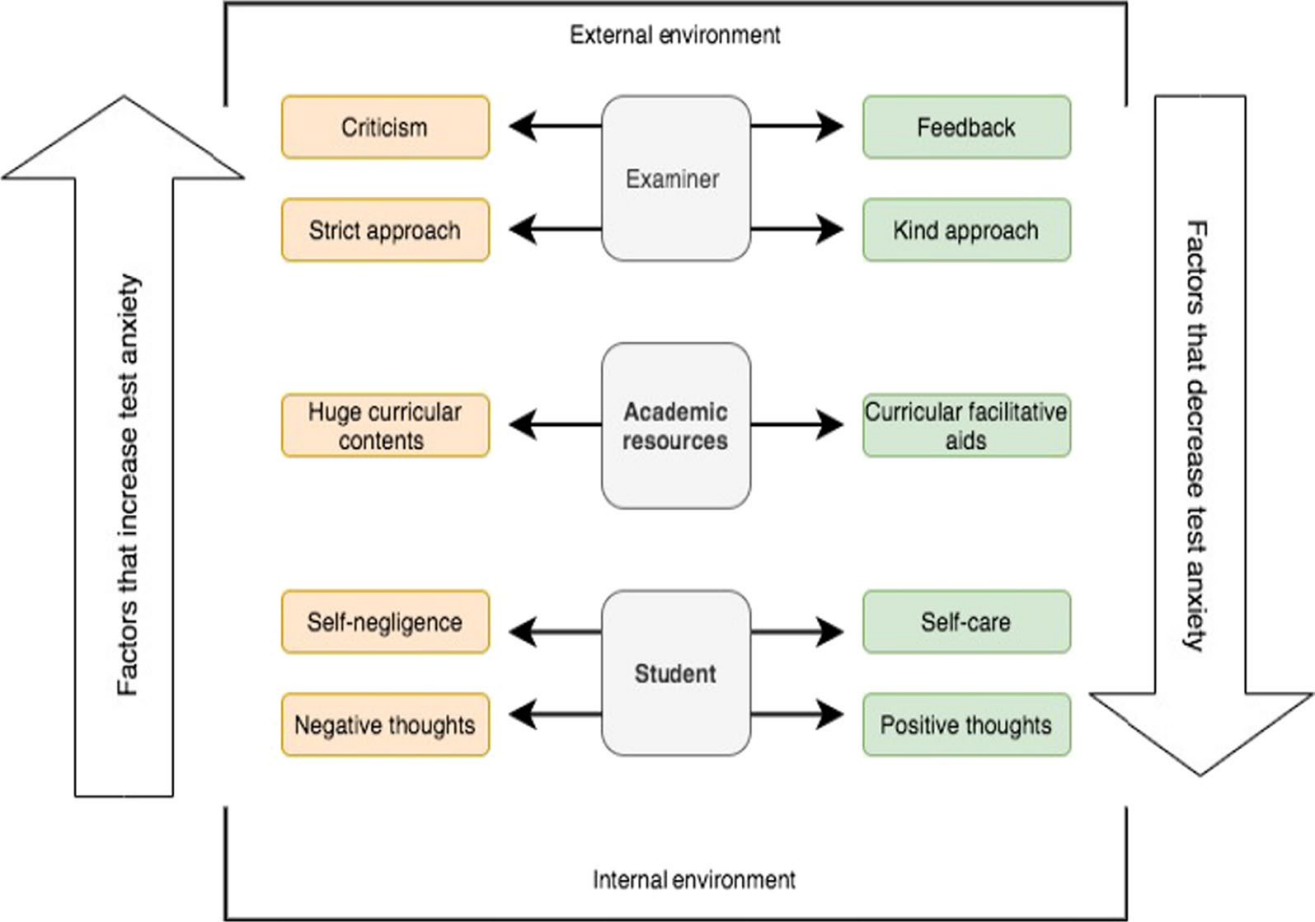
Emerged themes and sub-themes in relation to increasing and decreasing test anxiety

As depicted in Fig. 1, three major themes emerged from the thematic analysis: students, academic resources, and examiners. Each theme was subdivided into subthemes that reflected increased and decreased TA.

Shown in gray, the themes were placed at the center of the figure. All subthemes that increased TA were grouped together and colored red, while those that decreased TA were grouped together and colored green. The arrows on either side of the figure denote the two primary probe questions asked during the FGD. Notably, we arranged the themes and their associated subthemes from external to internal to reflect the relationships and interactions between them.

### Theme 1: students

**Negative vs. positive thoughts** Most students expressed how their negative and positive thoughts influenced TA:

Both [positively minded and negatively minded students] may make a mistake, but the positive [student] will go on with minor anxiety because they know what [is] done is done and they cannot change it, but the negative one will keep on dwelling [on] the past/mistakes, and hence [this student increases their] anxiety level. (Student E, Group 6)

Some students noted that their preconceptions of negative ideas occurred if they had to share a bad experience with their colleagues:

I [learned] that the malignant doctor will examine me the next day. Eight of the students failed. The day [that I learned this was] stressful[.] (Student B, Group 3)

When I [learn that a] “malignant doctor” will become my examiner, it increases [my] test anxiety[.] (Student C, Group 6)

Moreover, because the students identified as A-level students and had pursued their education since childhood, these expectations further increased their anxiety. Crucially, some students noted that positive thoughts and maintaining their motivation reduced TA:

I always try to keep myself positive and constantly remind myself to be confident in myself and believe in God. (Student A, Group 5)

My anxiety will decrease if I stay positive by […] telling myself that I will do well in the exam[.] (Student D, Group 2)

**Self-negligence vs. self-care** The more time that the students had for self-care and maintaining a healthy lifestyle, the more their positive thoughts increased. In particular, the students mentioned that adequate sleep, a balanced or fulfilling diet, and exercise lowered TA:

[W]hat helps me with stress or anxiety is good food and sleep[.] (Student C, Group 4)

For me [,] I need food to focus. For this reason, food relie[ves] my stress. (Student F, Group 5)

I tend to get anxious when I go [to take an] exam without having coffee and something to eat. (Student B, Group 3)

In contrast, self-negligence was associated with increased TA. The students reported many bad behaviors that impacted their health and exam preparedness:

I also tend to get anxious when I go for an exam without having coffee and something to eat[.] (Student E, Group 4)

I went to sleep at 7:00 am and [woke] up at 1:00 pm. I drink two cups of coffee per day. I [know that] some of my friends drink 5–10 cups of coffee [per day]. These kinds of things are not usual to your body. (Student B, Group 1)

### Theme 2: academic resources

**Heavy curriculum vs. facilitative curricular aids** The students pointed out that the heavy medical curriculum triggered their anxiety levels before their exams:

But the problem with medical school [is that] too [many things must] be prepared [for] before the exam[.] (Student D, Group 1)

The one that [stresses me out] is the [number of things] we need to cover […] for major exams especially[.] (Student C, Group 3)

I need to cover [so many things]. [All things] and [many things], and you do not have enough time[.] (Student A, Group 5)

Notably, supportive measures before the exam could ameliorate this burden. The suggested measures include increasing formative assessments, briefings on the exams’ formats,, and making class more fun:. In particular, the students emphasized the importance of formative assessments:

Give quiz[s]/homework based on[the] learning outline at the end of every lecture so the students know what exactly they have to cover for each subject[.] (Student A, Group 6)

In our previous exam, there [was] no briefing, so we [did not] know how many questions [would] come in the exam […] [or] what [would] be assessed[.] (Student C, Group 2)

Try to make the class more fun because fun and relaxing classes tend to increase one’s memory and focus during that class. To be honest, [the] lecturer[s]who make classes more fun and [use] more discussion tend to make me remember things easier. (Student A, Group 3)

### Theme 3: examiner

**Strict vs. kind approach** The students believed that the examiner played an important role in aggravating TA. Most stated that due to the presence of an examiner, OSCE was the assessment format that raised their TA the most:

[T]he examiner will affect me [the] most. (Student B, Group 4)

The examiner in OSCE is very strict about the answer scheme, and sometimes he/she is known as an examiner who always fail[s] the student[s]. (Student A, Group 2)

Most students agreed that the stricter the examiner, the worse their experience of TA. They identified some features of so-called “malignant examiners,” including their intention to fail students:

[My anxiety increases during the exam if] the examiner in OSCE is someone we know [who is] very strict about [the] answer scheme […] [or] known as an examiner who always fail[s] the student[s]. (Student B, Group 1)

Moreover, the students claimed that malignant examiners used certain facial expressions and body language during the exam:

[Examiners] who are grumpy and do not even answer back when I greet them make[] me more anxious during OSCE[.] (Student C, Group 1)

The [examiner] who has a straight face without any expression increases anxiety during exam[.] (Student A, Group 2)

Correspondingly, the students believed that kind examiners significantly reduced TA, such as by smiling and establishing a rapport:

I will choose the examiners that are kind and soft spoken to the student[s] to avoid the students feeling [scared] when [answering] the question[,] especially [during] OSCE[.] (Student C, Group 3)

Just a simple gesture such as a smile, talking nicely to student[s], [and focusing] on information given by students. All [of] these will help to reduce stress[.] (Student B, Group 6)

The students suggested that applying a unified scoring system will help in reducing TA:

I think [I would] I brief all examiners about the guidelines and make sure that examiners understand the guideline and what is actually expected of students[.] (Student E, Group 2)

[The examiners should] have a proper guideline for the marking of the students[.] (Student A, Group 4)

Everyone has a proper guideline, not given a bias of judgment. I think that is OK for me[.] (Student F, Group 3)

**Criticism vs. feedback** The students signified that the examiner’s response approach affected TA. Most participants described criticism as “scolding,” which increased TA:

I just fear […] being scolded during the exam. (Student A, Group 4)

I think that some examiners should not be shouty because student[s] are not well prepared. So, the way [that they] treat students will affect them. (Student D, Group 2)

Some […] examiners [scold students]. They usually compare [the] current situation with future work. Just [because] you are feeling stress now, this [does] not mean you cannot handle whatever [will come up] when you work. (Student D, Group 1)

Correspondingly, students agreed that giving effective and constructive feedback during exams reduced TA, supported their learning, and empowered them to prepare more for their upcoming exams:

[If the examiner feedbacks the student, he] will [be] happier[,] and after the exam[,] he will tell [his] other friends that [the] examiner [taught] him [well.](Student A, Group 1)

Actually, for both exams, I got the same marks. But, I was feeling better during the first exam, where the lecture calm[ed] me, and after the exam, she guided me [on how to answer] the question[.] [E]ven though I [could not] answer it, she guided me on what to do after that rather than scold[ing] me and ask[ing] me to [leave.] (Student B, Group 4)

## Discussion

This qualitative study explored TA from the perspective of medical students and identified its precipitating and diminishing factors: the students, their academic resources, and the examiner.

### Theme 1: students

**Negative vs. positive thoughts** The students had a major role in developing and depleting TA. This could be due to the nature of TA, which originates from a negative self-process that encodes the outer environment into personal responses. In this context, the process fixated on how the exam would be and how the examiners would interact with the examinees, and it was exaggerated by an individual’s negative self-thoughts, academic competence, and/or ability to cope with challenging evaluative situations. For example, many participants had developed self-expectations since childhood. These expectations were strengthened by the people surrounding them and continued to be enhanced during their medical education. Other studies have determined that parents’ academic expectations affect their offspring’s TA [20–22]. Notably, the participants in this study also worried about transmitting their bad experiences to their peers.

Our findings suggest that enriching positive thoughts and believing in self-efficacy reduces TA in medical students. In his investigation of the cognitive triad, Wong [23] proposed that rational beliefs will lead to neutral or positive emotional consequences. This assumption has been used by many researchers to build cognitive reconstructions to reduce TA [13, 24–27]. Based on these findings, it is apparent that thoughts influence our behavior [28]. Hence, we argue that negative and positive thoughts are the most important factors in this study. They may be used to form interventions to resolve TA.

**Self-negligence vs. self-care** The students demonstrated a variety of self-negligence behaviors that may amplify TA and trap them in a vicious cycle, including sleep deprivation, excessive consumption of coffee and other stimulants, and avoidance of sports. Self-negligence affects psychological perception and weakens mental fortitude, thus resulting in TA. On the other hand, self-care improves personality characteristics and the mental ability to combat TA [29, 30]. Hence, our results emphasize the critical state of self-care among medical students, and academic advisory programs, among others, should promote healthy lifestyle choices to rectify this.

### Theme 2: academic resources

Students can feel an exaggerated sense of pressure and increased TA if they have to face the difficulty of their medical curriculum from the beginning of their studies [9–12]. By incorporating facilitative curricular aids, the detrimental effects of the curriculum can be remedied. For example, classes should be enjoyable and employ more formative assessments. Another strategy is to promote group study, as this may establish a beneficial framework for discussing and illustrating ambiguous concepts. Group study has been shown to be an effective method for enhancing student learning and providing a welcoming environment for discussion [31].

### Theme 3: examiner

**Strict vs. kind approaches** According to socioconstructive theory and the social aspect of anxiety [32], the presence of an examiner (or evaluator) is the primary cause of TA during clinical examinations. The situation deteriorates further as a result of ineffective communication and attempts to fail students based on biases and prejudices. McManus et.al [33] referred to this approach as “the hawk effect” or “stringency.” As Shashikala [34] noted, such a person is occasionally referred to as the “malignant examiner.” Typically, examiners become stringent to ensure the principle of patient safety, which is the ultimate goal of medical education, during clinical evaluation of medical students. However, students’ mental well-being should not be jeopardized. For this reason, standardized scoring (either checklist or rubric) tools helps to eliminate prejudice and personal bias [35]. Nevertheless, comprehensive training on using the standardized scoring assessment during OSCE is highly mandated and crucial to maximize fairness and assessment validity [36].

**Criticism vs. feedback** The students noted that when they received constructive feedback during an exam, this enhanced their learning and helped them improve in a subsequent exam or practice session. Many students worried that some examiners may neglect the most effective use of feedback. Some examiners hope to motivate students by critically increasing their awareness and fear of failure, and Putwain and Roberts [34] referred to such tactics as “fear appeals.” The present study suggests that extensive training and close monitoring of the examiner during the examination will help reduce TA. Students should be encouraged to express themselves without harming the exam environment.

## Limitation

Two limitations apply to this study. The first is that it is restricted to a single medical school, and the second is that the sampling frame does not include representative medical students from all academic years, as medical students in their first and fifth years are required to sit for exams at the time of data collection. Both of these constraints make generalization difficult. For these reasons, additional research is needed to broaden the sampling and expand its scope beyond medical schools to include groups from other disciplines of health professions education (HPE). This will provide a comprehensive understanding of the TA problem across multiple HPE disciplines.

## Conclusion

This qualitative study shed light on the factors that affect TA from medical students’ perspectives. Three major factors affecting TA were identified: the students, their academic resources, and the examiner. Sub-factors were also identified for each of these factors. This study established a solid foundation for policymakers and decision makers in medical education to improve current assessment practices while also enhancing student well-being. The results indicate that the polar factors of the examiner and the student act in concert and shape TA. Thus, additional psychological support for students and training for examiners on how to deal with examinees will significantly reduce TA.

## Data Availability

Due to privacy concerns, the transcripts of the interviews are not available to the public. On reasonable request, the corresponding author can provide transcript information.

## Abbreviations

FGD: Focus group discussion
OSCE: Objective structured clinical examination
TA: Test Anxiety
USM: Universiti Sains Malaysia
USMLE: United States Medical Licensing Examination
